# rPredictorDB: a predictive database of individual secondary structures of RNAs and their formatted plots

**DOI:** 10.1093/database/baz047

**Published:** 2019-04-25

**Authors:** Jan Jelínek, David Hoksza, Jan Hajič, Jan Pešek, Jan Drozen, Tomáš Hladík, Michal Klimpera, Jiří Vohradský, Josef Pánek

**Affiliations:** 1 Department of Software Engineering, Faculty of Mathematics and Physics, Charles University, Ke Karlovu, Praha; 2 Laboratory of Bioinformatics, Institute of Microbiology, The Czech Academy of Sciences, Videnska, Praha; 3 Luxembourg Centre for Systems Biomedicine, University of Luxembourg, avenue du Swing, Belvaux

## Abstract

Secondary data structure of RNA molecules provides insights into the identity and function of RNAs. With RNAs readily sequenced, the question of their structural characterization is increasingly important. However, RNA structure is difficult to acquire. Its experimental identification is extremely technically demanding, while computational prediction is not accurate enough, especially for large structures of long sequences. We address this difficult situation with rPredictorDB, a predictive database of RNA secondary structures that aims to form a middle ground between experimentally identified structures in PDB and predicted consensus secondary structures in Rfam. The database contains individual secondary structures predicted using a tool for template-based prediction of RNA secondary structure for the homologs of the RNA families with at least one homolog with experimentally solved structure. Experimentally identified structures are used as the structural templates and thus the prediction has higher reliability than *de novo* predictions in Rfam. The sequences are downloaded from public resources. So far rPredictorDB covers 7365 RNAs with their secondary structures. Plots of the secondary structures use the Traveler package for readable display of RNAs with long sequences and complex structures, such as ribosomal RNAs. The RNAs in the output of rPredictorDB are extensively annotated and can be viewed, browsed, searched and downloaded according to taxonomic, sequence and structure data. Additionally, structure of user-provided sequences can be predicted using the templates stored in rPredictorDB.

## Introduction

Currently, RNA secondary structures are either identified experimentally or computationally predicted. While experimental identification is technically too demanding to be routinely used, computational prediction in general cannot predict accurate individual RNA structures.

Available databases of RNA structures follow a similar trade-off between structure quality and the amount of structures available; PDB (1) contains experimentally identified structures of individual RNAs. Most of the structures in PDB are reliable and PDB provides so far the best source of the RNA structures, but the scope of PDB is extremely limited. Similarly, CRW (2), which is dedicated to ribosomal RNAs (rRNAs), contains a combination of experimentally identified and manually adjusted structures that are reliable, but cover only rRNAs. More extensive, but still limited to solved structures, is STRAND (3), an RNA secondary structures database that collects known secondary structures of RNAs from all sources and all types. Rfam (4) on the other hand provides predicted consensus secondary structures; it is much less reliable than either PDB or CRW, but its scope is extensive. rPredictorDB aims to be a middle ground between PDB and Rfam—it strives to provide structures that are more reliable than those in Rfam but its scope is significantly wider than both PDB and CRW.

In rPredictorDB, we provide secondary structures of selected individual RNAs. The structures are predicted using experimentally identified structures as templates that are transferred to related sequences with a previously published method (5). We use this approach primarily because the secondary structures generated by the template-based prediction are, in comparison with the *de novo* predicted ones, more biologically reliable (5). Our method also provides individual secondary structures that are easier to use in downstream analysis, including identification, comparison, visualization and functional analysis of RNAs—in contrast to consensus structures as stored in Rfam that may require further processing and specialized tools. The experimentally identified structural templates and also the sequences of the homologous RNAs for which the secondary structure is generated are acquired from public databases and literature. The predicted secondary structures are periodically updated to follow changes in the original sources and stored in our database.

The stored secondary structures are visualized using RNAplot (6) and Traveler (7). Traveler is a tool for template-based visualization of RNA secondary structures. In rPredictorDB, it is applied to visualize the secondary structures of RNAs with large structures that are hard to be visualized clearly with the standard tools.

Available RNAs are phylogenetically sorted and can be searched by keyword and sequence and browsed by taxonomy for exploration, comparison, identification and visualization purposes. rPredictorDB also provides extensive documentation available at its website.

## rPredictorDB architecture

**Figure 1:**
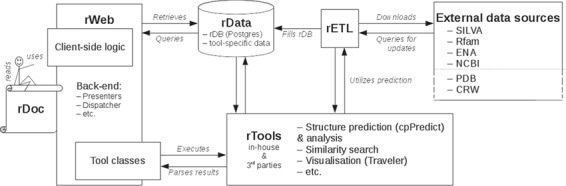
rPredictorDB architecture.

rPredictorDB consists of the following components: a set of tools that perform standard tasks on the data such as similarity search, secondary structure prediction and visualization of the predicted secondary structures (rTools); a database of RNA sequences, their predicted secondary structures and annotations (rData); extraction–transformation–load mechanisms to build rData (rETL); and a web-based frontend (rWeb) that provides easy access to rData and rTools for the community. The overall architecture of rPredictorDB is depicted in Figure 1. In the following sections we will describe the individual components of rPredictorDB.

### Secondary structure prediction (rTools)

RNA secondary structure prediction methods are reviewed in Fallmann *et al*. (8). Most commonly, RNA secondary structure prediction is performed using free energy minimization by dynamic programing techniques [reviewed in (9)] and comparative methods [reviewed in (10)]. The quality of former depends strongly on both the sequence length and the type of the predicted structure. The latter requires multiple homologous sequences that may not be available, depend on the chosen homologous sequences and the multiple sequence alignment technique. Moreover, the resulting consensus structure is not always representative of the structures of individual RNAs as the consensus structure can differ substantially from the individual ones.

To avoid shortcomings of those approaches, rPredictorDB employs template-based prediction of RNA secondary structures as described in Panek *et al*. (2017) (5). Briefly, the method uses known secondary structures of different, but homologous, molecules as the structural templates. It identifies conserved and unconserved subsequences within an RNA molecule; for conserved subsequences, the template structure is directly transferred into the generated structure and combined with *de novo* predicted structure of the unconserved subsequences. The method also determines when the generated structure is unreliable.

Evaluation of the reliability of the generated structures is based on a bootstrap of the tree edit distance between the template and the generated structure of the query sequence with hundred times randomly shuffled dinucleotides. The reliability is evaluated in the form of z-score included in the rPredictorDB output page. The z-scores values >2 indicate reliable generated secondary structure, while the z-scores values <2 indicate that the template and the query RNA are not homologous.

The template-based method is advantageous especially for large secondary structures, whose prediction is inefficient and/or unreliable with the classical prediction methods. Examples are large rRNAs (~ 1500–5400 nucleotides) with complex structure (which are included in rPredictorDB).

### Visualization of RNA secondary structures (rTools)

In rPredictorDB, secondary structures are visualized using two methods: RNAplot (6) and Traveler (7). The first method is used as a standard for visualization of secondary structures of all RNAs. Traveler, an algorithm for template-based visualization of RNA secondary structures, is employed to obtain readable, systematically laid out plots of RNAs with long sequences.

Since human-authored layouts for secondary structure of large RNA molecules are mostly habitual, it is difficult to come up with an algorithm that would mimic such layouts. The approach taken by Traveler is similar to the one described in the previous section for the template-based prediction of RNA secondary structures. However, unlike for prediction, the input to the algorithm consists of a target structure and a template structure together with the known layout. Traveler then converts both the target and the template structures into their tree representations and applies tree edit distance to obtain mapping between the structures. The mapping is then used to modify the template layout to transfer it on the target one. Such a template-based approach is especially useful with rRNA structures that are, due to evolutionary reasons, highly conserved and not many modifications of the template layout are required to adapt it to the target structure. The resulting plots can be colored to highlight residues which were modified, inserted or reinserted due to substantial modification of the respective secondary structure.

### Data sources

The structure templates required for the prediction algorithm are mined manually from public databases and literature. To avoid propagating shortcomings and biases of available prediction algorithms, we only consider experimentally determined structures. rPredictorDB currently includes 36 templates for 36 RNA families. Of the 36 templates, 4 were found in literature, 1 in SRPDB and the remaining in PDB. To our knowledge, these are all RNA structures currently available in PDB. The templates are listed in Table 1 along with their sources. The templates were extracted from their sources using RNApdbee (11) and manually adjusted to resolve problems in conversion.

**Table 1:** RNA families included in rPredictorDB and their templates and sources. Names of RNAs are derived from names of PDB structures

**RNA**	**Source of sequences**	**Templates and their source**	**Template sequence length (nucleotides)**
16S rRNA		*Escherichia coli* 16S rRNA (PDB ID 2ZM6)^e^	1542
18S rRNA *Chordata*	SILVA	*H. sapiens* 18S rRNA (PDB ID 4V6X)^e^	1869
18S rRNA *Diptera*		*Drosophila melanogaster* 18S rRNA (PDB ID 4V6W)^e^	1995
5S rRNA *Bacteria*	Rfam (RF00001)	*E. coli* 5S (PDB ID 1C2X)^e^	120
5S rRNA *Eukarya*		*H. sapiens* 5S (PDB ID 6EKO)^e^	120
5.8S rRNA	Rfam (RF00002)	*Trypanosoma cruzi* 5.8S RNA (PDB ID 5T5H)	169
6S	Rfam (RF00013)	*E. coli* 6S (17)^b,d^	184
		*Bacillus subtilis* 6S RNA (18)^b,d^	187
9S rRNA	Rfam (RF02545)	*Trypanosoma brucei* 9S rRNA (PDB ID 6HIY)	621
Cobalamin riboswitch	Rfam (RF00174)	*Symbiobacterium thermophilum* (PDB ID 4GXY)	172
C-DI-AMP riboswitch	Rfam (RF00379)	*Thermovirga lienii* C-DI-AMP riboswitch (PDB ID 4QK9)	123
CRPV-IRES	Rfam (RF00458)	Mammalian CRPV-IRES (PDB ID 6D9J)	190
CSFV IRES	Rfam (RF00209)	Viral CSFV IRES (PDB ID 4C4Q)	233
FMN riboswitch	Rfam (RF00050)	PDB ID 3F2Y^f^	112
Fungi U3	Rfam (RF01846)	*Saccharomyce cerevisiae* u3 (PDB ID 5WYK)	333
gcvB	(19)^b^	*Salmonella typhimurium* gcvB (19)^b^	206
GLMS ribosyme	Rfam (RF00234)	*Bacillus anthracis* GLMS ribosyme (PDB ID 3L3C)	141
Group I catalytic intron	Rfam (RF00028)	*Staphylococcus* virus Twort (PDB ID 1Y0Q)	192
Group II intron lariat	NCBI^a^	*Oceanobacillus iheyensis* group II intron (PDB ID 5J02)	418
Group II intron lariat in post-catalytic state^c^	NCBI^a^	*Pylaiella littoralis* (PDB ID 6CIH)	621
IRES HCV	Rfam (RF00061)	*H. sapiens* IRES HCV (PDB ID 5A2Q)	257
Lariat capping ribozyme	Rfam (RF01807)	*Didymium iridis* lariat capping ribozyme (PDB ID 4P8Z)	188
Lysine riboswitch	Rfam (RF00168)	*Thermotoga maritima* lysine riboswitch (PDB ID 4ERL)	161
Mammalian CPEB3 ribozyme	Rfam (RF00622)	*H. sapiens* CPEB3 (20,21)^b^	78
M-box	Rfam (RF00380)	*B. subtilis* M-box (PDB ID 3PDR)	161
micF	Rfam (RF00033)	*E. coli* micF (22)^b^	95
MLV encapsidation signal	Rfam (RF00374)	Viral MLV (PDB ID 1U6P)	101
ms1	(23)^b^	*Mycobacterium smegmatis* ms1 (23,24)^b^	304
oxyS	Rfam (RF00035)	*E. coli* oxyS (25)^b^	109
PHI29 PROHEAD RNA	Rfam (RF00044)	Bacteriophage PHI29 (PDB ID 1FOQ)	117
RNaseP arch	Rfam (RF00373)	*Pyrococcus furiosus* RNaseP (26)	347
RNaseP bact a	NCBI^a^	*Thermoanaerobacter tengcongensis* RNaseP bact a (PDB ID 3Q1R)	347
RNaseP bact b	Rfam (RF00011)	PDB ID 2A64^f^	414
RNaseP nuc	Rfam (RF00009)	*H. sapiens* RNaseP (27)^b^	341
ryhB	(28)^b^	*E. coli* ryhB (28))^b^	90
SAM I	Rfam (RF00162)	*T. tengcongensis* SAM I (PDB ID 2GIS)	94
spot42	Rfam (RF00021)	*E. coli* spot42 (29)	119

(Continued)

**Table 1:** Continued

**RNA**	**Source of sequences**	**Templates and their source**	**Template sequence length (nucleotides)**
SRP bact small	Rfam (RF00169)	*E. coli* SRP (SRPDB ID esccol3d-97-11-17-stretched.pdb)	114
SRP bact large	Rfam (RF01854)	*B. subtilis* SRP (PDB ID 4UE4)	266
SRP Metazoa	NCBI^a^	*H. sapiens* SRP (PDB ID 4P3E)	301
Tetrahymena ribozyme	NCBI^a^	PDB ID 1X8W^f^	247
*Tetrahymena* TR	Rfam (RF00025)	*Tetrahymena* TR (PDB ID 6D6V)	159
THF riboswitch	Rfam (RF01831)	PDB ID 4LVV^f^	89
tmRNA	Rfam (RF00023)	*E. coli* tmRNA (PDB ID 3IZ4)	377
TPP riboswitch	NCBI^a^	*E. coli* TPP (PDB ID 4NYG)	83
tRNA Gly eukaryotic	Rfam (RF00005)	*H. sapiens* tRNA Gly (PDB ID 5E6M)^e^	74
tRNA Gly bacterial		*Geobacillus kaustophilus* tRNA Gly (PDB ID 4MGM)^e^	75
u2	Rfam (RF00004)	*H. sapiens* u2 (30)^b^	188
u1	Rfam (RF00003)	*H. sapiens* u1 (30)^b^	163
u4	Rfam (RF00015)	*H. sapiens* u4 (31)^b^	144
u5	Rfam (RF00020)	*H. sapiens* u5 (32,33)^b^	116
u6	Rfam (RF00026)	*H. sapiens* u6 (PDB ID 5LQW)	112
Vertebrate TR	Rfam (RF00024)	*H. sapiens* TR (16)^b^	451
Yeast u1	Rfam (RF00488)	*S. cerevisiae* PDB ID 5ZWN	565

^a^ The sequences were obtained by NCBI BLAST search with ‘somewhat similar sequences’ parameters against nr database with query sequences taken from PDB. The reason was that the sequences in an appropriate Rfam family seemed incompatible with PDB structure, as they either were short fragments or had very low sequence similarity to the PDB sequence.

^b^ Sequences and/or template structure were copied from the paper publishing the template structure.

^c^ This family contains several very short fragments producing substructures that are hard to match with the template structure. Nevertheless, we included them into rPredictorDB as they had significant BLAST e-values (<1.10^−12^) and also, as they represent a good example of RNAs with extremely fragmented sequences.

^d^ It is impossible to distinguish which template should be used based on taxonomy, as some bacteria, e.g. *Firmicutes*, contain 6S RNAs of both template types. Therefore, the template producing a structure with a better z-score is used for each 6S RNA.

^e^ The template is applied to sequences according to taxonomy, i.e. a eukaryotic template to eukaryotic sequences, a prokaryotic template to prokaryotic sequences.

^f^ Organism not described or a synthetic expression system used.

In order to make the predicted secondary structures more readable, we provide human-authored layouts for the templates. Layouts for rRNAs were downloaded from CRW and manually edited. For the rest of the templates that were not laid out well with RNAplot, we manually rearranged the structure plots using VARNA (12).

The sequences for which we predict secondary structure were extracted from SILVA database (13) and Rfam (14) with additional metadata taken from ENA (http://www.ebi.ac.uk/ena) and NCBI Taxonomy (https://www.ncbi.nlm.nih.gov/taxonomy). rPredictorDB currently includes 53 RNA families with a total of 7365 sequences. The mapping between the templates and families in Rfam/categories in SILVA is maintained manually.

For most rRNAs, SILVA contains numerous sequence variants and/or fragments of the same variants. They produce strongly similar or identical structures or substructures of the same structure. In addition, rRNAs are strongly evolutionarily conserved and therefore their structures repeat or are very similar even for more evolutionarily distant species. If we included them all into rPredictorDB, we would overload it by rRNAs. Therefore, we included a single, representative RNA with the longest sequence with highest quality for each 18S rRNA subspecies and each 16 rRNA family. If the rRNA requested by a user is not in rPredictorDB, the user has the option to let rPredictorDB predict it using an appropriate rRNA template.

### Extraction—transformation—load (rETL)

To import the sequence and the annotation data into rDB, rPredictorDB uses an ETL layer called rETL. The core part of stored data—the RNA primary structures (nucleotide sequences) and their unique accession number identifier are extracted straightforwardly from SILVA and Rfam. More care is, however, needed for sequence metadata. Additional fields available from SILVA are the sequence quality measures (SILVA is well curated in this respect and has a comprehensive sequence quality control system). SILVA also provides taxonomic information for the sequences, but this information is not always correct (e.g. salmon and alligator classified as Mammalia) and Rfam does not provide taxonomic information, so this information is added later manually to ensure consistency. ENA provides a wealth of additional annotation about the sequence: references to scientific literature, classification by source molecule type, the scientific name, method of obtaining the sequence, etc. ENA also contains taxonomic information; however, this information is sometimes incomplete or even completely missing. Also, in some cases alternative names are used (e.g. some ‘Diptera’ are classified as ‘Endopterygota’ and some are classified as ‘Holometabola’). To avoid these ambiguities, we take a taxonomic path for corresponding scientific name from NCBI Taxonomy. It is also checked that the taxonomic path from NCBI contains all taxons or their synonyms mentioned in the taxonomic information from ENA.

**Figure 2:**
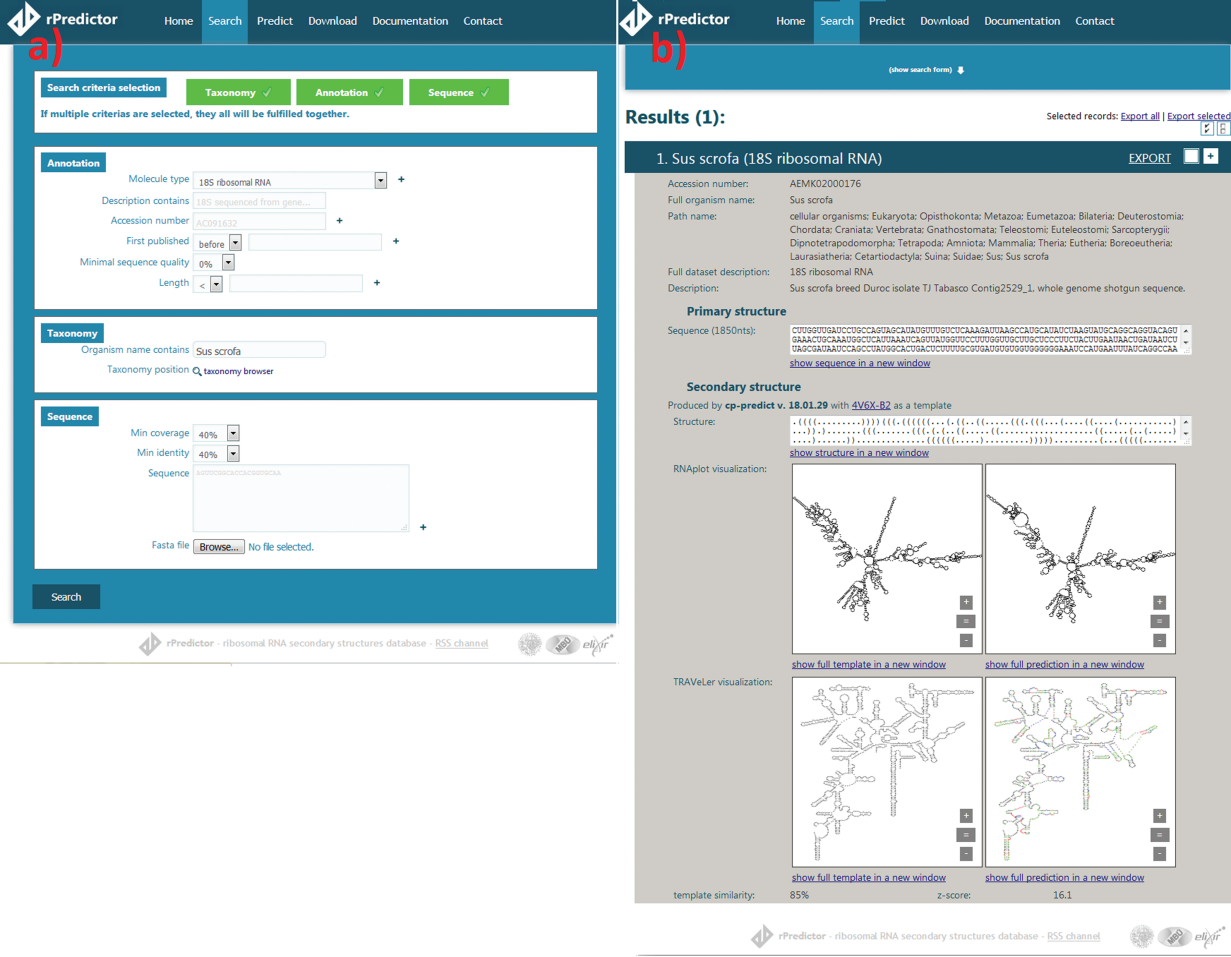
Snapshots of the rPredictorDB input (a) and output (b) interface. The searched RNA is *Sus scrofa* 18S rRNA.

The secondary structures of the downloaded sequences are then generated automatically using the stored templates. In addition to the generated structures, a list of structural features is identified for each generated structure. The structural features are secondary structure motifs including hairpin loops, internal loops, junctions, 5′ and 3′ overhangs and others. After prediction, all of the prepared data are stored in the database. The ETL step is run periodically to keep up to date with the original sources. For further details of the ETL process, including the complete ETL schema, see the online documentation.

## Web-based interface

The presentation component of rPredictorDB is called rWeb. It is a web application developed in PHP with Nette framework (https://nette.org/). The frontend uses JavaScript and jQuery scripting library (https://jquery.com/) that provides access to the search and prediction modules.

**Figure 3:**
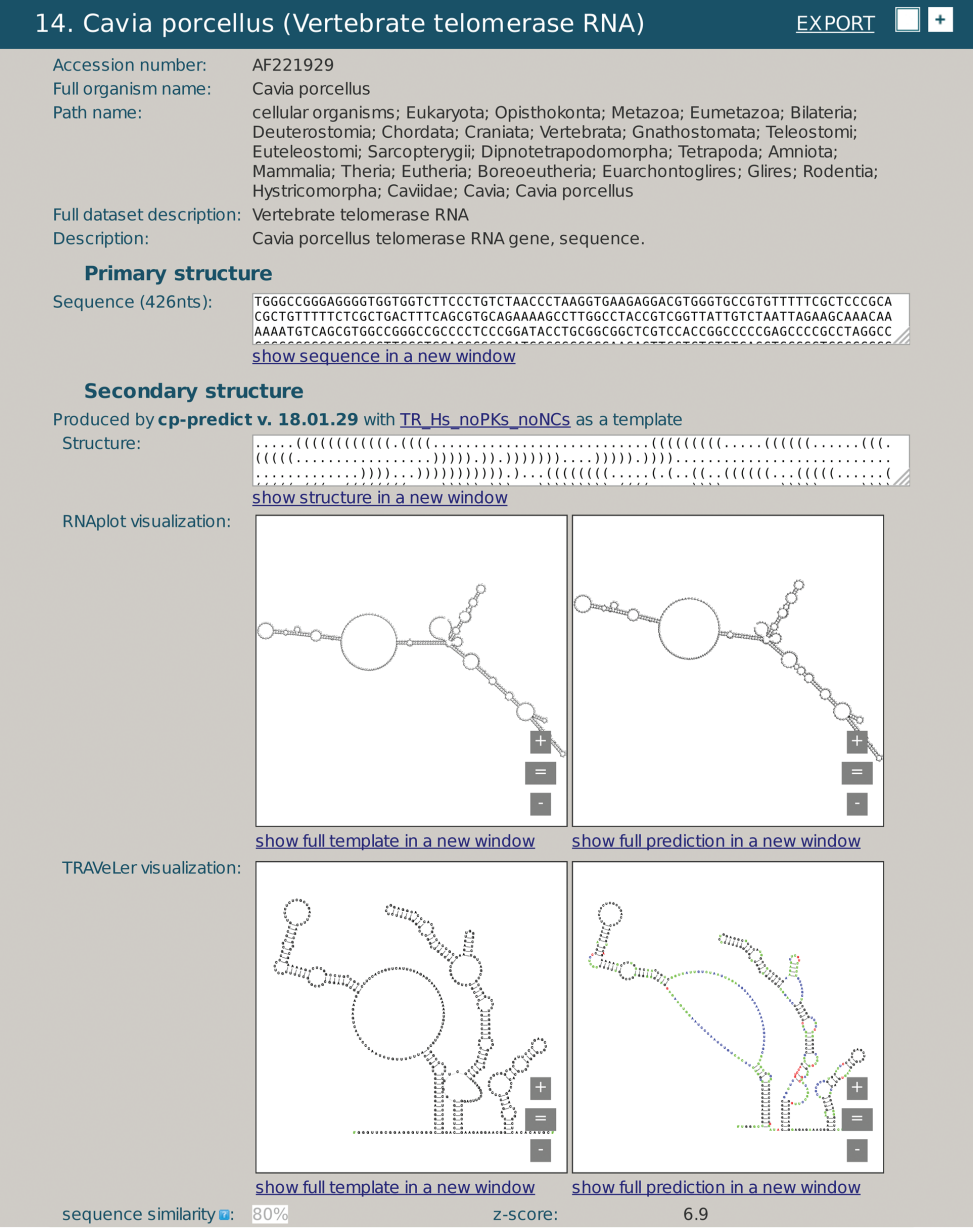
A snapshot of rPredictorDB output for *C. porcelanus* TR RNA. Panels (a) and (c) show secondary structure of a template (*H. sapiens* TR RNA) displayed by RNAplot and Traveler, respectively. Panels (b) and (d) show secondary structure of *C. porcelanus* TR predicted using *H. sapiens* TR RNA as a template, displayed by RNAplot and Traveler, respectively.

The search module allows users to search by sequence similarity, annotation, taxonomy and other criteria that can be specified at the same time (Figure 2a). Sequence search allows users to input sequence(s) they want to search for in the rDB using BLAST+ (15) together with minimum coverage (a length of a matched fragment relative to a length of the input sequence) and identity (a relative number of matches in the alignment) constraints. Coverage and identity filters were chosen instead of e-value as they allow a finer filtering—for example, in the case of long 18S rRNA, e-value is often zero even for more distant alignments like that between *Lutzomyia toroensis* and *Emphysomera conopsoides* with 58% coverage and 86% identity. Searching by annotation allows the user to search by molecule type, ENA description, ENA accession number, sequence quality (in the case of sequences originated from SILVA) or length. Finally, searching by taxonomy lets the user to either specify the scientific name of the required organism or to use the taxonomy browser to search for a taxon by browsing the phylogenetic tree.

The records matching the search criteria are then presented to the user showing the annotations and the primary structure, the generated secondary structure and the visualization generated by Traveler (7)—if necessary—or RNAplot (6) (Figure 2b). The search results can be exported into CSV, JSON or FASTA format.

The user is also given the option to predict secondary structure for an uploaded RNA sequence(s) using one of the templates stored in rPredictorDB. If the option ‘Select template automatically’ is on, a sequence alignment between the query and all templates is performed using BLAST+ and the template with the most similar sequence is used. The output of the secondary structure prediction includes the generated secondary structure in dot-bracket notation, its plot and the measures of similarity between the query and the template (Figure 2c).

Finally, rPredictorDB provides the option to download the whole rDB either as a PostgreSQL database dump or as a CSV export.

Detailed information on the rPredictorDB web-based interface can be found in the rPredictorDB documentation available at its website.

## Example usage of rPredictorDB

**Figure 4:**
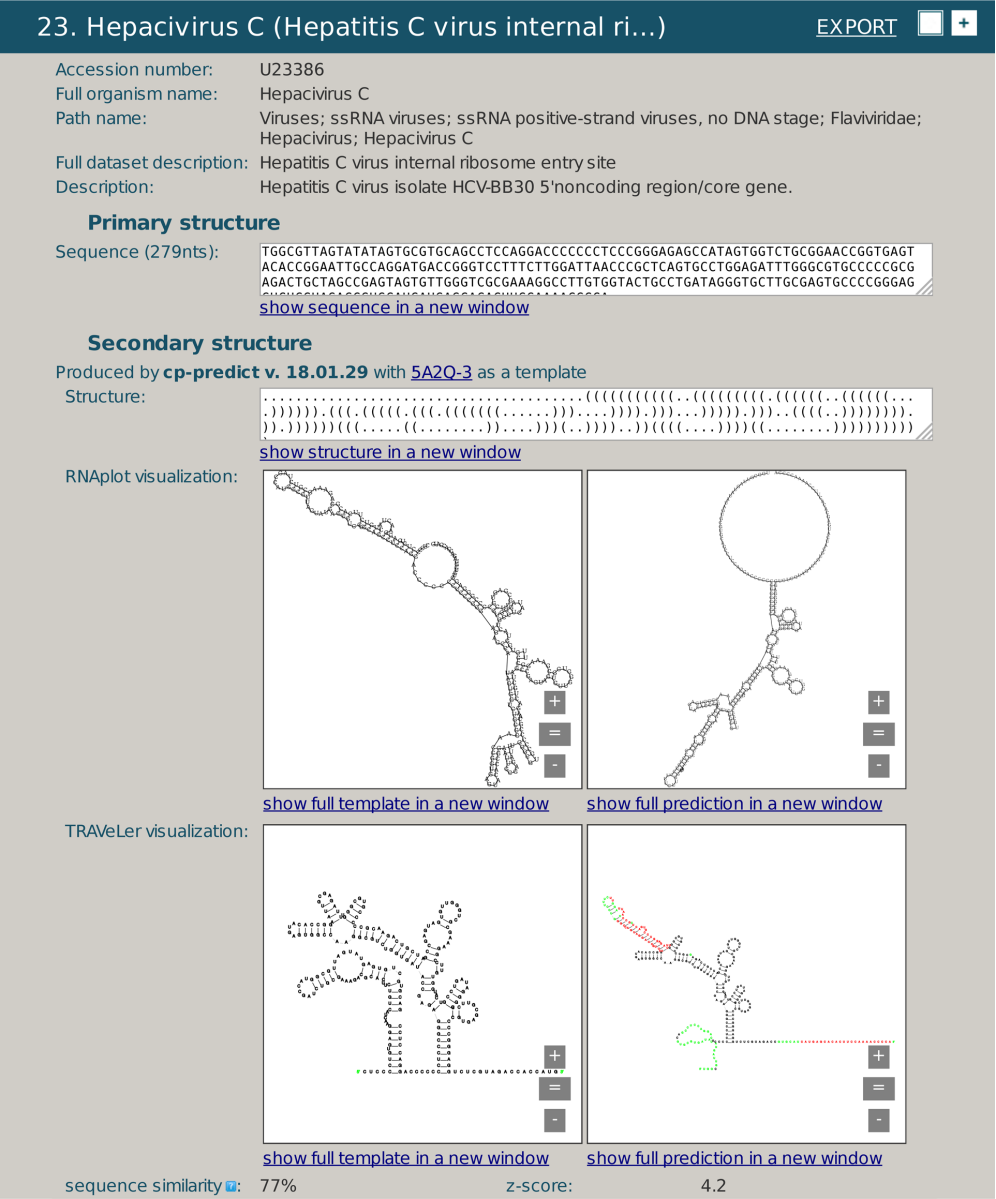
A snapshot of rPredictorDB output for *Hepacivirus C* IRES HCV RNA. Panels (a) and (c) show secondary structure of the template (*Hepacivirus C* IRES HCV RNA PDB ID 5A2Q) displayed by RNAplot and Traveler, respectively. Panels (b) and (d) show secondary structure of *Hepacivirus C* IRES HCV RNA with acc. # U23386 predicted using *Hepacivirus C* IRES HCV RNA PDB ID 5A2Q as a template, displayed by RNAplot and Traveler, respectively.

We demonstrate the use of rPredictorDB by searching for two of the RNAs included in it, the vertebrate Telomerase RNA (TR) (Figure 3) and IRES HCV RNA (Figure 4). In the first example, the secondary structure of *Cavia porcelanus* TR (Figure 3b) was generated using *Homo sapiens* TR structure (16) as the template (Figure 3a).

Both the template and the generated secondary structures were plotted by RNAplot (Figure 3a and b). More readable visualization is achieved using formatted plots, as shown in Figure 3c and d for the template and the generated structure, respectively. The plots make it possible to present the secondary structures clearer, and they are visually better comparable and allow for more detailed inspection. The plots also show differences between the templates and the generated structures using colors. Green color denotes relabeled nucleotides, red color encodes inserted nucleotides and blue is used for nucleotides that had to be repositioned due to the substantial modification of the corresponding loop or a hairpin.

The example shows only one RNA, but there are in total 37 vertebrate TRs from various organisms stored in rPredictorDB that we have omitted for brevity. Nevertheless, one of the aims of rPredictorDB is the possibility to list homologs of a single RNA together with both their sequence and the reliable secondary structure accompanied by the annotations that can be used for comparison, identification, visualization and other purposes.

The second example shows the secondary structure generated by rPredictorDB for a fragment of *Hepacivirus C* IRES HCV RNA (Figure 4b) using 5A2Q PDB structure as the template (Figure 4a). The comparison of the template and the generated structure shows which substructure is coded by the fragment (cf. Figure 4a and b). The comparison is more efficient using the formatted plots (cf. Figure 4c and d) that also show differences in both the sequence and the structure marked by different colors (Figure 4d). This example demonstrates one of the uses of rPredictorDB for identification of fragmented sequences of homologous RNAs using their secondary structures.

Note, that as in the previous example, this structure is only one of all (79) IRES HCV RNAs included in rPredictorDB. Similar analysis can be done for each of the RNAs.

## Results and discussion

We present rPredictorDB, a database of predicted secondary structures of individual RNAs. It uses template-based prediction to generate secondary structures using experimentally identified RNA structures as templates. The visualization of the generated structures is improved by a template-based visualization method.

Currently, rPredictorDB stores 7365 RNAs of 53 RNA families. For each RNA, its predicted secondary structure and annotation are available. To our knowledge, these 53 RNA families comprise a comprehensive, although possibly incomplete list of the RNA families with at least one experimentally identified structure.

The RNA families in rPredictorDB do not necessarily match the Rfam families, as they can be formed based on sequences sources other than Rfam, e.g. papers publishing biochemically probed RNA structures together with sequences of their homologs, NCBI sequences found by BLAST or the SILVA database.

So far, we do not to include RNAs with sequences shorter than 100 nucleotides into rPredictorDB to keep it aimed primarily at RNAs with ‘longer’ sequences and complex structures. The structures of the rRNAs with shorter sequences (≤100 nucleotides) can be relatively accurately predicted using available tools, or these RNAs have reliable consensus structures in Rfam. Using these RNAs, we think we would just copy Rfam. The RNAs with sequences < 100 nucleotides that are currently in rPredictorDB are for testing purposes of the user interface of the web server.

In rPredictorDB, the user can input his/her own sequence(s) to obtain its secondary structure generated by template-based prediction using one of the templates in rPredictorDB. The template with the sequence most similar to the query sequence is used. The reliability of the secondary structure generated by rPredictorDB is reported by its z-score. If there is no homologous template available in rPredictorDB, the generated secondary structure will not be biologically reliable and its z-score will be <2. This option is included in rPredictorDB as to help to analyze unidentified RNA sequences.

By the presented rPredictorDB approach, we try to overcome disadvantages of consensus structures stored in Rfam. To that end, we employ the template-based prediction that was shown previously to be more reliable than other prediction methods including the one based on refold.pl (5).

However, rPredictorDB depends on the availability of experimentally identified structures. In principle, their use as templates for prediction of structures of other rRNAs is biologically meaningful only for homologous RNAs that have the same function and thus have similar structures. The content of the rPredictorDB database is therefore restricted to the RNA families with at least one member with an experimentally solved structure, but still has much wider coverage than either PDB or CRW.

As the number of experimentally solved RNA structures grows with the improvement of the techniques of structural biology we think that rPredictorDB will be increasingly useful for the analysis of RNAs including their identification, comparison and visualization.

## Availability

rPredictorDB is freely available at http://rpredictor.ms.mff.cuni.cz/.

## Notes

**Database URL:** http://rpredictordb.elixir-czech.cz
